# Curcumin Induces Autophagy, Apoptosis, and Cell Cycle Arrest in Human Pancreatic Cancer Cells

**DOI:** 10.1155/2017/5787218

**Published:** 2017-09-10

**Authors:** Yaping Zhu, Shurui Bu

**Affiliations:** Department of Gastroenterology, Jinshan Hospital Affiliated to Fudan University, 1508 Lonhang Road, Jinshan District, Shanghai 201508, China

## Abstract

**Objective:**

Curcumin is an active extract from turmeric. The aim of this study was to identify the underlying mechanism of curcumin on PCa cells and the role of autophagy in this process.

**Methods:**

The inhibitory effect of curcumin on the growth of PANC1 and BxPC3 cell lines was detected by CCK-8 assay. Cell cycle distribution and apoptosis were tested by flow cytometry. Autophagosomes were tested by cell immunofluorescence assay. The protein expression was detected by Western blot. The correlation between LC3II/Bax and cell viability was analyzed.

**Results:**

Curcumin inhibited the cell proliferation in a dose- and time-dependent manner. Curcumin could induce cell cycle arrest at G2/M phase and apoptosis of PCa cells. The autophagosomes were detected in the dosing groups. Protein expression of Bax and LC3II was upregulated, while Bcl2 was downregulated in the high dosing groups of curcumin. There was a significant negative correlation between LC3II/Bax and cell viability.

**Conclusions:**

Autophagy could be triggered by curcumin in the treatment of PCa. Apoptosis and cell cycle arrest also participated in this process. These findings imply that curcumin is a multitargeted agent for PCa cells. In addition, autophagic cell death may predominate in the high concentration groups of curcumin.

## 1. Introduction

Pancreatic cancer (PCa) is the third leading cause of cancer-related death in the United States with a 5-year survival rate of 7.7% [1] and ranks 12th of all cancer incidences. Nearly 81% PCa patients were diagnosed at a terminal stage, which determines a poor prognosis. According to some statistical data, both morbidity and mortality of PCa continue to rise, while those of most other cancers have declined [2]. Surgery is applicable only to a few early-stage patients, and chemotherapy is the most important remedy for patients with metastatic cancer. Pre- and postoperative chemotherapy can also benefit the patient. According to a randomized study [3], the median survival increased from 4.41 months of treatment with 5-FU to 5.65 months of gemcitabine, and the 1-year survival rate improved from 2% in 5-FU-treated patients to 18% in gemcitabine-treated patients. Gemcitabine has therefore become the first-line chemotherapy regimen. However, owing to multidrug resistance and the intolerable adverse effects of the drug, searching for new alternative and adjuvant chemotherapy drugs has become an urgent mission.

The notion of autophagy was put forward several decades ago to describe the “self-eating” phenomenon extensively existing in many organisms [4]. It is a process of degrading cytoplasmic ingredients especially protein reacting against harsh conditions like nutrition deficiency and stress. Recent evidence suggests that autophagy is a double-edged sword in tumorigenesis and metastasis, because it can suppress tumor formation but on the other hand it promotes tumor growth once the tumor is formed [5]. Some studies [6, 7] demonstrated that autophagy inhibition could attenuate PCa activity markedly. mTOR (mechanistic target of rapamycin) possesses serine/threonine kinase and acts as an important regulator of cellular growth and metabolism [8]. In normal conditions, mTOR always represses the ULK1-Atg13-FIP200 complex and blocks autophagy, while autophagy ensued when mTOR activity was suppressed in the state of nutritional scarcity or stress [5, 9]. This pathway can trigger the formation of autophagosomes. At the same time, LC3-I is formed by the removal of the C-terminal 22 amino acids from LC3, followed by a conversion of some LC3-I into LC3-II, leading to the maturity and isolation of autophagosomes. The amount of LC3-II has a positive correlation with the extent of autophagosome formation and thus can be regarded as a good marker of autophagy [10].

Curcumin, as an ingredient of turmeric, has attracted increasing attention for decades due to its various biological effects, including anti-inflammatory [11], antioxidant [12], and anticancer [13] properties. Curcumin is extracted from the rhizome of curcuma longa belonging to the ginger family and chemically known as 1,7-bis-(4-hydroxy-3-methoxyphenyl)-hepta-1,6-diene-3,5-dione, with the chemical formula of C_21_H_20_O_6_ [13, 14]. Numerous experiments in vitro and vivo have demonstrated that curcumin could inhibit the growth of various cancers including gastric cancer, ovarian cancer, and colorectal cancer by inducing apoptosis [15–17] or curbing cell proliferation individually [18]. In addition, some studies in recent years have shown that autophagy plays a certain role in the anticancer process of curcumin [19–21]. However, the underlying mechanism remains elusive and controversial. The aim of the present study was to determine whether autophagy plays a role in the treatment of PCa with curcumin and explore the underlying mechanism.

## 2. Materials and Methods

### 2.1. Cell Lines and Reagents

PANC1 and BxPC3 cell lines were the subjects of this study as representatives of human pancreatic cancer cell lines, where PANC1 cells were derived from ductal epithelial cells and BxPC3 cells were derived from acinous adenocarcinoma. On the other hand, human-derived cell lines are closer to clinical drug efficacy than animal sources. Both of them were purchased from Xiangya Cell Center of the Central South University (Changsha, China). PANC1 cells were cultured in Dulbecco's modified Eagle's medium (DMEM) and BxPC3 cells in RPMI 1640 medium at 37 °C in 5% CO_2_. Both of them were supplied with 10% fetal bovine serum (FBS). Curcumin (P0206, purity > 98%, purchased from PureOne Biotechnology, Shanghai, China) was formulated into liquid at 100 mg/ml with DMSO, followed by being diluted into different required concentrations with culture medium as follows.

### 2.2. Cell Growth Inhibition Test

Cells were seeded in 96-well plates with 10^4 cells per well. Curcumin with a concentration gradient was added in different groups after cells adhered to the bottom. At first, PANC1 cells were treated with curcumin at concentrations of 0, 0.2, 2, 10, 20, 40, 80, and 200 *μ*g/ml for 24 h. It was found that there were no differences in cell morphology and proliferative status at the concentration of 0, 0.2, 2, and 10 *μ*g/ml, but when the concentration was 20 *μ*g/ml, the cell proliferation began to be inhibited and the cell morphology changed from the regular spindle and polygonal shape to round shape, and the intercellular antennae began to be reduced. With the increase of culture concentration, cell debris gradually increased and cell proliferation was inhibited obviously. When the incubated concentration reached 200 *μ*g/ml, there was almost only cell debris left. So the concentrations of 0, 10, 20, 40, and 80 *μ*g/ml were selected to study the cell proliferation rate for PANC1 cells, and similarly, BxPC3 cells were treated with 0, 0.4, 0.8, 1, 4, 8, 10, and 20 *μ*g/ml of curcumin. The incubation time was set at 24, 48, and 72 h. Cell viability was tested via Cell Counting Kit-8 (CCK-8, DOJINDO, Japan) with absorbance at 450 nm.

According to the results, the groups were set as follows: 0, 10, 20, 40, and 80 *μ*g/ml of curcumin for PANC1 cells and 0, 0.1, 0.5, 1, 5, and 10 *μ*g/ml for BxPC3 cells in the following experiments, among which 0 *μ*g/ml group was the control group. The incubation time was set to 24 hours.

### 2.3. Cell Cycle Analysis

Cells of different groups as previously described were harvested and washed with PBS and fixed with 75% ethanol at 4°C overnight. After being stained with PI/RNase solution (BD Pharmingen, 550825) for 15 min in the dark, cells cycle distribution of different groups was detected by flow cytometry (Becton Dickinson, USA).

### 2.4. Apoptosis Quantitative Detection

The two cell lines were trypsinized after being treated with different concentrations of curcumin as previously described and washed three times with PBS. The operating procedures were performed referring to the instruction. Cells were double stained with PI (Propidium Iodide)/Annexin V-FITC kit (DOJINDO, Japan). All the samples were detected using a BD flow cytometer. The apoptosis quantity was defined as the sum of the Q2 and Q3 quadrant. Data and diagrams were treated with FlowJo 7.6 software.

### 2.5. Immunofluorescent Assay

In this study, we used LC3 to detect autophagy. After being treated with different concentrations of curcumin in 24-well plates, cells were fixed with paraformaldehyde for 15 min and incubated with LC3 antibody (Cell Signaling Technology, #12741) overnight. The next day, the second antibody conjugating with FITC (a fluorescent dye) was used to detect LC3 with a fluorescent microscope.

### 2.6. Western Blotting Test

Protein obtained by lysis of cells, sonication, and centrifugation procedurally [22] was loaded to each well, electrophoresed in polyacrylamide gels, and transferred onto the polyvinylidene difluoride (PVDF) membrane. After being blocked with 5% nonfat milk for 1 h, membrane was incubated with primary antibodies overnight at 4°C, including anti-LC3 (1 : 1000), anti-caspase 3 (1 : 1000, CST, #9663), anti-mTOR (1 : 1000, CST, #2983), anti-Bax (1 : 1000, CST, #2772S), anti-Bcl2 (1 : 1000, CST, #2870S), anti-*β*-actin (1 : 5000, KeyGEN BioTECH, KGAA001, Jiangsu, China), and anti-*α*-Tubulin (1 : 1000, CST, #2144S). Then, the membrane was incubated with second antibodies (goat anti-rabbit IgG-HRP) for 1 h at room temperature on a shaker. Finally, bands were visualized via ECL assay (KeyGEN BioTECH, Jiangsu, China) by the Tannon 5200 automatic imaging system (Shanghai, China), and optical density (OD) was measured by Tannon GIS image analysis system.

### 2.7. Statistical Analysis

Correlation analysis between cell viability and LC3II/Bax was calculated by SPSS 22.0. Other statistical analysis results were completed by GraphPad Prism 5 software. One-way analysis of variance and Bonferroni's multiple comparison test were used to determine differences between groups. *p* value < 0.05 was considered statistically significant.

## 3. Results

### 3.1. Curcumin Inhibits the Proliferation of PCa Cells

**Figure 1:**
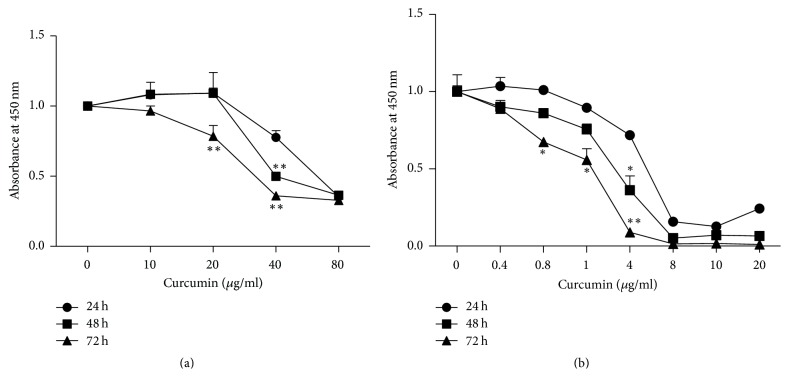
Curcumin inhibits the proliferation of PCa cells in a time- and dose-dependent manner. (a) As cultivate time and concentration increase, the cell viability deteriorates for PANC1 cell line. (b) Similar inhibitory effect was found in the BxPC3 cell line. Data are representative of thrice independent experiments. Error bar, SEM. *∗* and *∗∗*, compared with 24 h group. ^*∗*^*p* < 0.05. ^*∗∗*^*p* < 0.01.

The inhibitory effect of curcumin on the PCa cell lines showed a dose- and time-dependent trend. As shown in Figure 1, the absorbance at 450 nm of the ordinate represents cell viability. As for PANC1 cells (Figure 1(a)), cell viability of 40 *μ*g/ml in 48 h and 72 h groups was significantly lower than 24 h group, and cell proliferation of 20 *μ*g/ml in 72 h group was inhibited more than 24 h group. What is more, with the increase of drug concentration, cell viability decreased gradually at each incubation time group. Curcumin exerted the similar dose- and time-dependent inhibitory effect on BxPC3 cells (Figure 1(b)). They were 4 *μ*g/ml group at 48 h and 0.8, 1, and 4 *μ*g/ml groups at 72 h that showed significant differences compared with 24 h groups, respectively. The higher the concentration of curcumin, the lower the cell viability.

### 3.2. Curcumin Induces Cell Cycle Arrest in PCa

**Figure 2:**
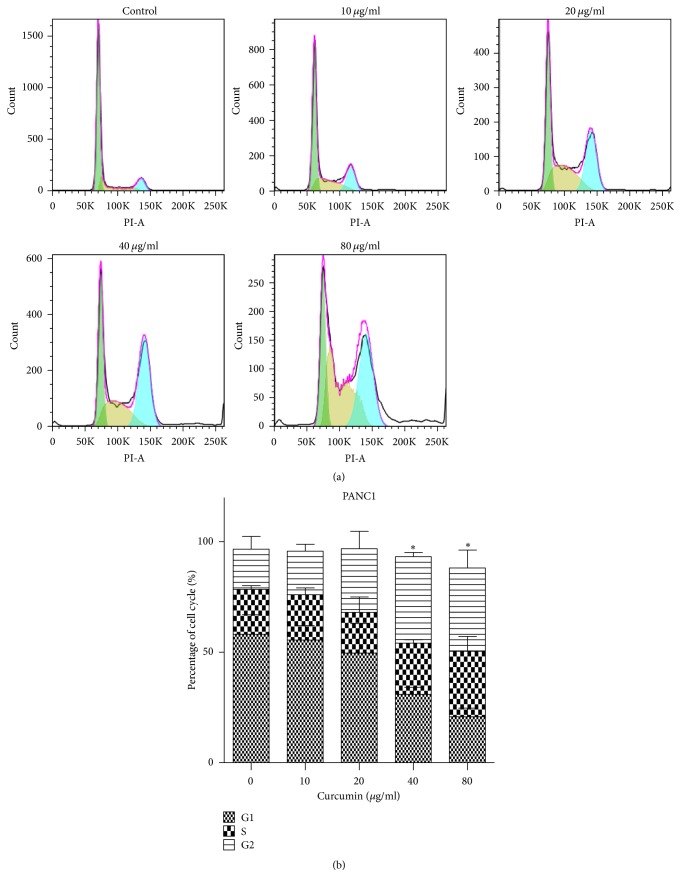
Curcumin induced G2/M arrest in PANC1 cell line. (a) Cell cycle distribution was detected by flow cytometer. (b) The proportion of G2/M stage obviously increased in 40 and 80 *μ*g/ml groups. Each dataset represents three independent experiments. Error bar, SEM. *∗*, significantly different from control group (*p* < 0.05).

**Figure 3:**
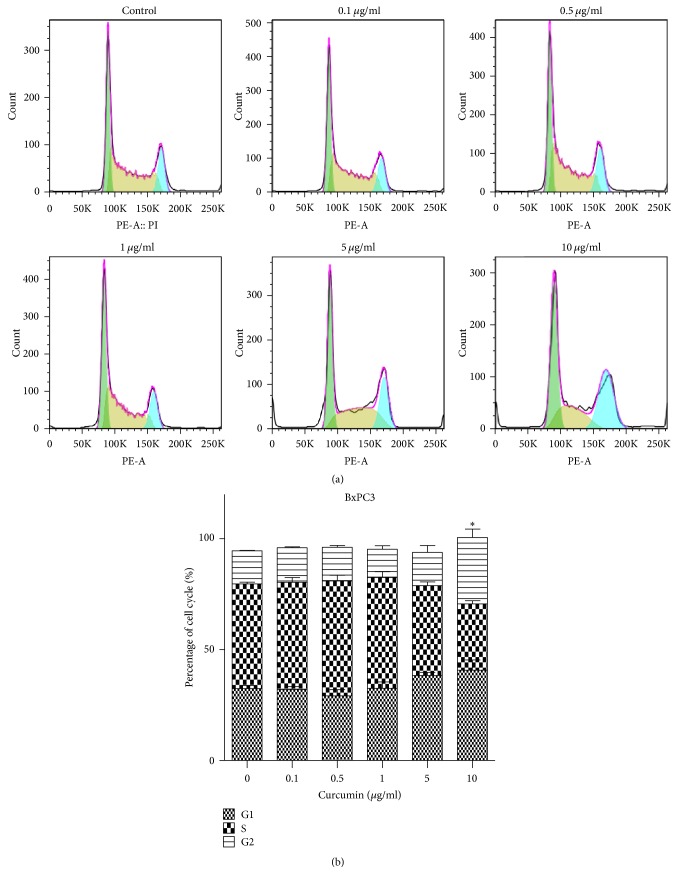
Curcumin induced G2/M arrested in BxPC3 cells. (a) Cell cycle distribution of BxPC3 cells after being treated with different concentration of curcumin. (b) The proportion of G2/M stage significantly increased in 10 *μ*g/ml group. Data are representative of three independent experiments. Error bar, SEM. *∗*, significantly different from control group (*p* < 0.05).

As previously described, PANC1 cells were treated with curcumin of 0, 10, 20, 40, and 80 *μ*g/ml for 24 hours, and BxPC3 cells were cultured with 0, 0.1, 0.5, 1, 5, and 10 *μ*g/ml of curcumin for 24 h. PCa cells cycle was arrested at G2/M stage (Figures 2 and 3). The average proportion of G2/M stage in 0, 10, 20, 40, and 80 *μ*g/ml groups was 18.1%, 19.6%, 28.8%, 39.1%, and 37.6% for PANC1 cells orderly (Figure 2). Compared with control group, cell cycle in 40 and 80 *μ*g/ml groups was significantly blocked (Figure 2(b)). The average proportion of G2/M stage for BxPC3 cells in 0, 0.1, 0.5, 1, 5, and 10 *μ*g/ml groups was 14.9%, 15.6%, 15.2%, 12.7%, 15%, and 29.9%, successively (Figure 3). 10 *μ*g/ml group had a significantly difference compared with the control group (Figure 3(b)). The results suggested that curcumin blocked PCa cells in G2/M phase.

### 3.3. Curcumin Induces Apoptosis of PCa Cells

#### 3.3.1. Effect of Curcumin on Cell Apoptosis Detected by Flow Cytometer

**Figure 4:**
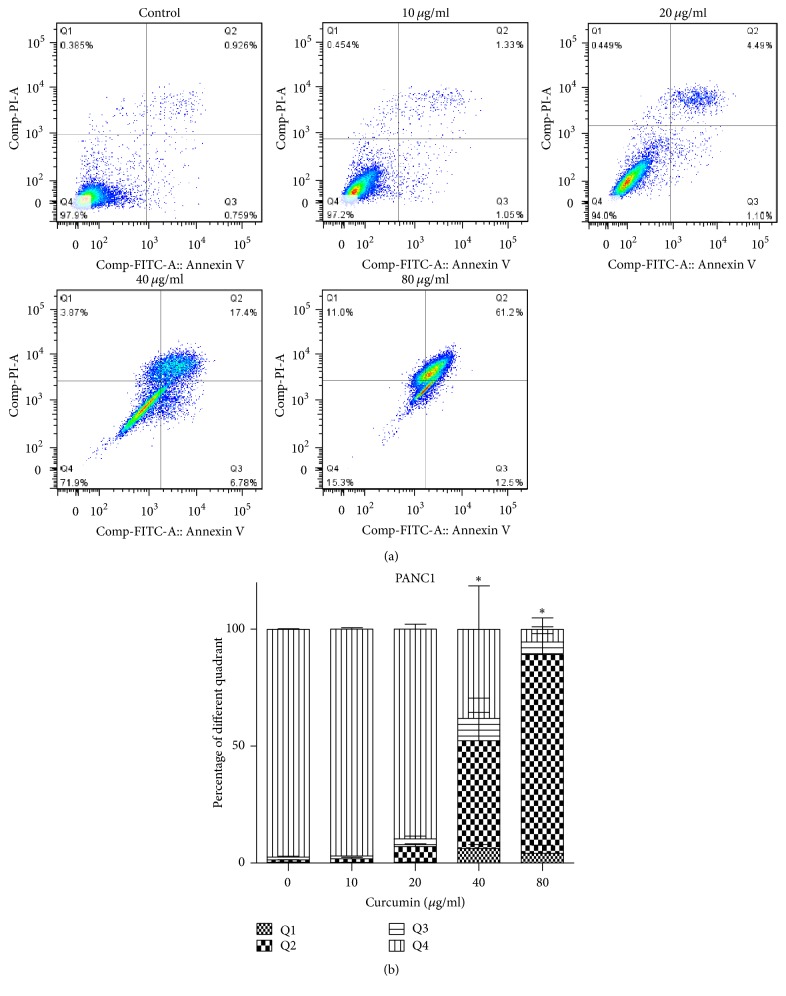
Curcumin induced apoptosis in PANC1 cells, measured by flow cytometry. (a) Each diagram was composed of four parts on behalf of different living state of cells. The total ratio of lower right (Annexin V^+^/PI^−^) and upper (Annexin V^+^/PI^+^) quadrants stands for apoptosis level. (b) The percentage of apoptosis was significantly higher in 80 *μ*g/ml groups. Data are representative of three independent experiments. Error bar, SEM. *∗*, significantly different from control group (*p* < 0.05).

**Figure 5:**
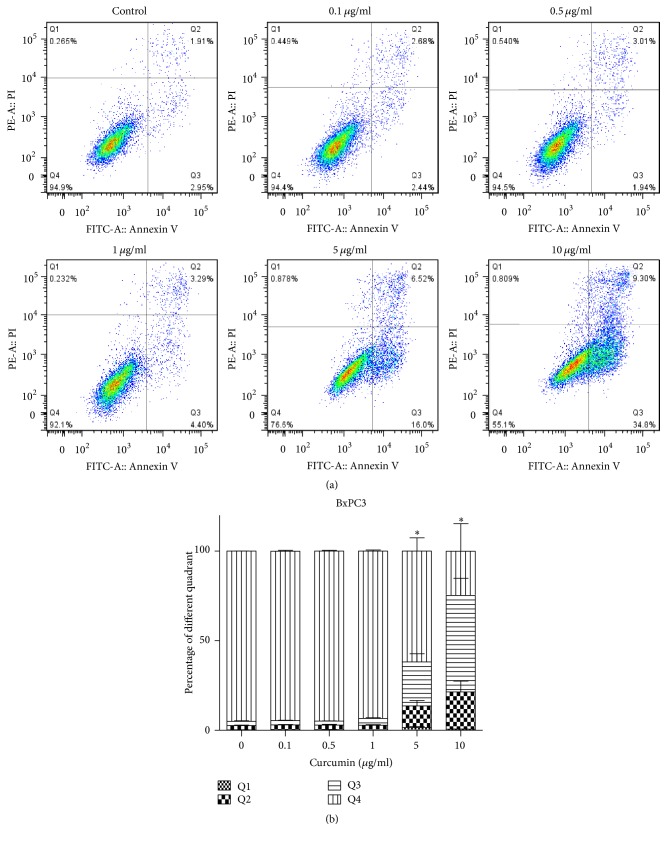
Curcumin induced apoptosis in BxPC3 cell lines. (a) Percentage of vitality, apoptosis, and necrosis was detected by flow cytometer. (b) The total ratio of apoptosis (Q2 and Q3 quadrants) was higher in 10 ug/ml groups. Data are representative of three independent experiments. Error bar, SEM. *∗*, significantly different from control group (*p* < 0.05).

The apoptosis ratio of 0, 10, 20, 40, and 80 *μ*g/ml groups for PANC1 cells was 2.3%, 2.8%, 9.9%, 55.6%, and 90.3%, orderly (Figure 4). The total ratio of lower right (Annexin V^+^/PI^−^) and upper (Annexin V^+^/PI^+^) quadrants which revealed that apoptosis level was higher in 80 *μ*g/ml group than control group (Figure 4(b)). As for BxPC3 cells, the apoptosis ratio of 0, 0.1, 0.5, 1, 5, and 10 *μ*g/ml groups was 4.7%, 5.1%, 4.8%, 6.5%, 36.6%, and 74.6%, respectively (Figure 5). And it is 10 *μ*g/ml group that had significant difference from control group (Figure 5(b)). Consistent with cell cycle distribution image, we can see the apoptotic peak in 40 and 80 *μ*g/ml groups of PANC1 cells and 5 and 10 *μ*g/ml groups of BxPC3 cells.

#### 3.3.2. Effect of Curcumin on Bax/Bcl2 Protein Expression

**Figure 6:**
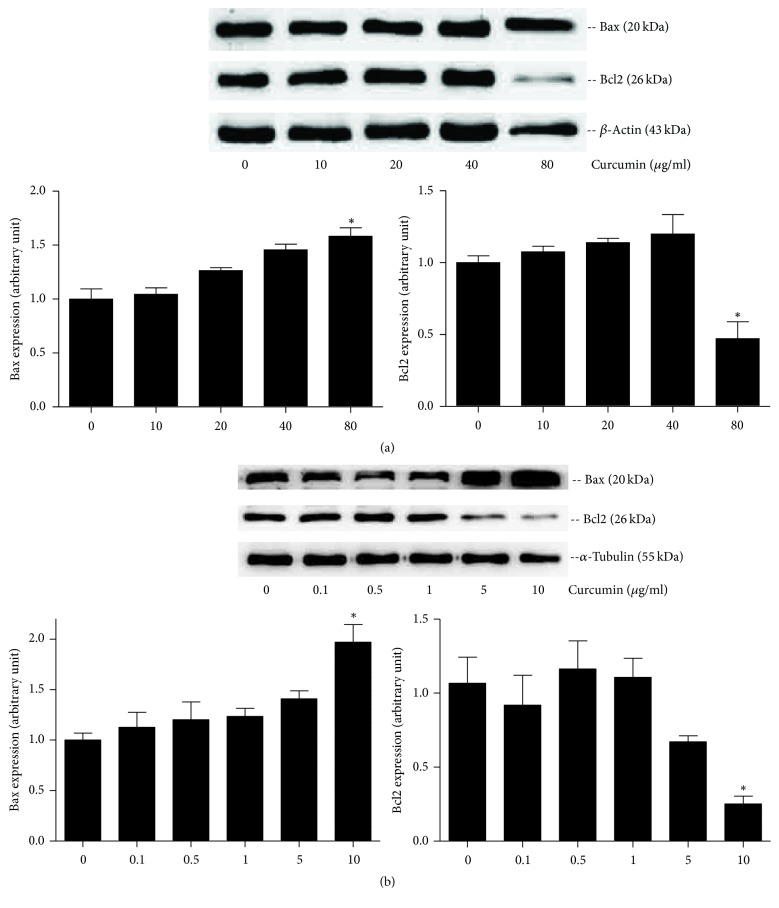
Expression of Bax and Bcl2 protein in PCa cells, detected by Western blot assay. (a) PANC-1 cells were treated with curcumin of different concentration. The protein expression of Bax was significantly increased in 80 *μ*g/ml group, while Bcl2 protein expression was decreased in 80 *μ*g/ml group. (b) The protein expression of Bax was significantly increased in 10 *μ*g/ml group of BxPC3 cells, while Bcl2 protein expression was decreased in 10 *μ*g/ml group. Error bar, SEM. *∗*, significantly different from control group (*p* < 0.05).

Bax is one of proapoptosis regulators [23], and Bcl2 belongs to the antiapoptosis molecular family [23, 24], both of which have long been used to detect the level of apoptosis. In this study, protein expression of Bax was significantly increased in 80 *μ*g/ml group for PANC1 cells (Figure 6(a)) and 10 *μ*g/ml group for BxPC3 cells (Figure 6(b)) compared to untreated control groups. In addition, Bcl2 protein expression was significantly decreased in 80 *μ*g/ml group for PANC1 cells (Figure 6(a)) and 10 *μ*g/ml group for BxPC3 cells (Figure 6(b)).

### 3.4. Curcumin Induces Autophagy of PCa Cells

#### 3.4.1. Curcumin Increases Autophagosomes in PCa Cells

**Figure 7:**
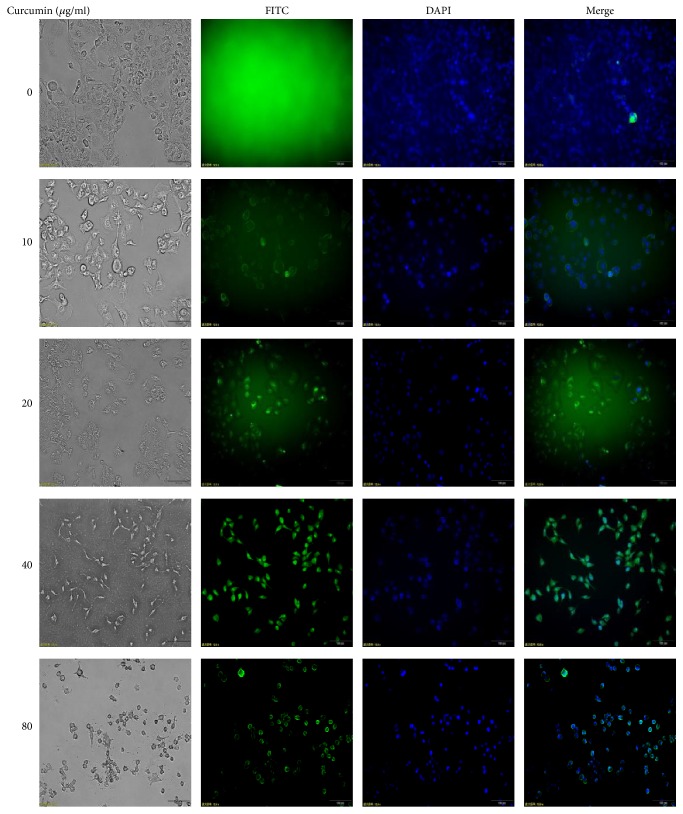
Autophagosomes in PANC1 cells were detected by immunofluorescence method. Cells were stained with FITC dye connecting second antibody after being incubated with LC3 antibody for an hour. Positive green dots in dosing groups show more when compared with the control group.

**Figure 8:**
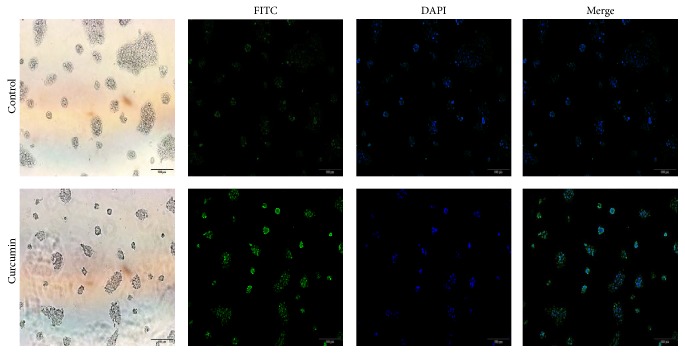
Immunofluorescence micrographs of BxPC3 cell lines by staining LC3 protein. Positive green-light puncta were detected in BxPC3 cells incubated with curcumin at a dose of 1 *μ*g/ml for 24 h versus negative in the blank control group.

In this study, luminescent autophagosomes were probed by tracking LC3 protein in cell immunofluorescence way (Figures 7 and 8). It was found that the expression level of punctuate autophagosomes was the highest in 40 *μ*g/ml group for PANC1 cells (Figure 7), and control group did not present much green autophagosomes. Similarly, positive green-light puncta were also detected in BxPC3 cells incubated with curcumin at a dose of 1 *μ*g/ml for 24 h versus negative in the blank control group (Figure 8).

#### 3.4.2. Effect of Curcumin on LC3II and mTOR Protein Expression

**Figure 9:**
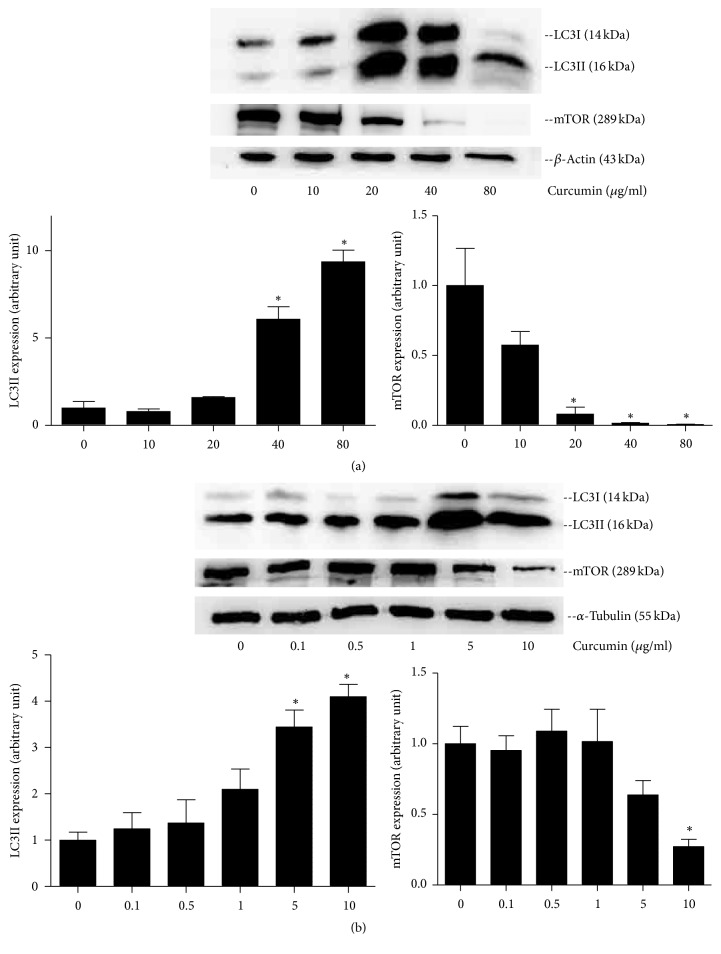
Expression of LC3II and mTOR protein in PCa cells, detected by Western blot assay. (a) PANC-1 cells were treated with curcumin of different concentration. The protein expression of LC3II was significantly increased in 40 and 80 *μ*g/ml groups, while mTOR protein expression was decreased in 20, 40, and 80 *μ*g/ml group. (b) The protein expression of LC3II was significantly increased in 5 and 10 *μ*g/ml groups of BxPC3 cells, while mTOR protein expression was decreased in 10 *μ*g/ml group. Error bar, SEM. *∗*, significantly different with control group (*p* < 0.05).

The protein expression of LC3II was significantly increased in 40 and 80 *μ*g/ml groups for PANC1 cells (Figure 9(a)). Similarly, the protein expression of LC3II was significantly increased in 5 and 10 *μ*g/ml groups of BxPC3 cells. In addition, the result of Western blot showed that the expression of protein mTOR was reduced significantly when cells were exposed to curcumin, especially at a dose of 20, 40, and 80 *μ*g/ml for PANC1 cells and 10 *μ*g/ml for BxPC3 cells (Figure 9). These results are consistent with the previous conclusion that the downregulation of mTOR could trigger the appearance of autophagosomes accompanied with the formation and flow of LC3II [25, 26].

#### 3.4.3. Comparison between Apoptosis and Autophagy

**Figure 10:**
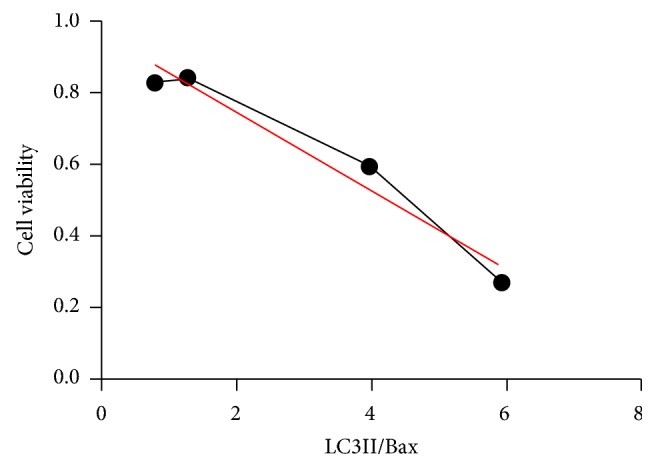
Correlation analysis between cell viability and LC3II/Bax. *r* = −0.979, *p* < 0.05. The greater the ratio, the lower the cell viability.

In this study, we calculated the relative ratio of Bax and LC3II (Table 1) and did the correlation analysis between cell viability and LC3II/Bax (Figure 10). With Pearson method, we got the results that *r* value equaled −0.979 and *p* value was less than 0.05. It meant that the greater the ratio, the lower the cell viability. In addition, when the incubation concentration of curcumin was high, like 80 *μ*g/ml group, the ratio of LC3II protein was far much higher than the ratio of Bax protein.

## 4. Discussion

Pancreatic cancer is a malignant tumor with a poor prognosis despite surgical intervention, and the standard chemotherapy is based on gemcitabine. However, the mild efficacy of gemcitabine against the drug-resistant pancreatic cancer limits its application. In this study, we found that curcumin had a diverse range of targets for PCa cells, including cell cycle arrest, apoptosis, and autophagy pathways. The multitargeted characteristics can make curcumin act synergistically in combination with standard chemotherapy drugs [27], especially for drug-resistant cancers.

Curcumin, a spice commonly used in curries and other south Asian cooking, has been found to possess anticancer effects, including breast cancer, colorectal cancer, lung cancer, and PCa [28–30], which is consistent with the results obtained in this study. Curcumin was found to inhibit the growth of two PCa cell lines in a dose- and time-dependent manner (Figure 1), and BxPC3 cells were more sensitive to curcumin compared with PANC1 cells (Figure 1). In this study, we found that curcumin could induce cell cycle arrest at G2/M stage in high concentrations (40 and 80 *μ*g/ml for PANC1 cells and 10 *μ*g/ml for BxPC3 cells) (Figures 2 and 3). It has been previously described that gemcitabine can induce cell cycle arrest in the S phase [31, 32]. Curcumin may produce synergistic effects when combined with gemcitabine by total arrest of cell cycle in most phases. Further studies are needed to confirm this presumption.

Apoptosis, also called programmed cell death, is a complex process of metabolic mediation involving numerous molecules in the organism. The amplified apoptosis results in damage or death of cells and tissues. While tumors will take place as apoptosis is weakened, in some sense, the antitumor mechanism of many chemotherapy drugs is to promote apoptosis. Similar results were obtained with curcumin in PCa cells. Previous experiments reported that treatment of BxPC3 cells with curcumin caused significant cell arrest in the G2/M phase and induced significant apoptosis [33]. In this study, curcumin was found to induce apoptosis of PANC1 and BxPC3 cell lines with the culture concentration increasing (Figures 4(a) and 5(a)), and the apoptosis fraction in high concentration groups (80 *μ*g/ml curcumin for PANC1 cells and 10 *μ*g/ml for BxPC3 cells) was significantly higher than that in the control groups (Figures 4(b) and 5(b)). Furthermore, the expression of proapoptosis protein Bax was significantly upregulated in high concentration groups (80 *μ*g/ml for PANC1 cells and 10 *μ*g/ml for BxPC3 cells), and the expression of antiapoptosis protein Bcl2 was downregulated in the same groups (Figure 6), demonstrating that curcumin promoted apoptosis of PCa cells.

Autophagy is a self-help process that provides cells with necessary amino acids by degrading damaged organelles or proteins against harsh conditions, stress, or hypoxia conditions [34]. Recent findings suggest that curcumin can induce autophagy to suppress proliferation of cancer cells as a prodeath or inhibitory signal [19, 35, 36]. Few studies have focused on the autophagy of PCa cells by curcumin. In this study, we demonstrated that curcumin could promote autophagosome formation in PCa cells (Figures 7 and 8). Then, curcumin was found to obviously upregulate the expression of LC3II protein (Figure 9) and downregulate the expression of mTOR protein (Figure 9). These results demonstrate that curcumin could induce the autophagy of PCa cells.

Finally, we compared the relative intensities of apoptosis and autophagy in different concentrations of curcumin by quantifying the Bax and LC3II protein expression (Table 1). We found that there was a negative correlation between cell viability and the LC3II/Bax ratio (Figure 10). We therefore speculated that autophagy-related cell death may play a key role in the high concentration of curcumin, and further experiments need to be carried out to test it. The signaling pathways and molecules of apoptosis and autophagy are widely interconnected, and many efforts have also been made to illustrate the interplay between them [37, 38]. When the level of stimulation is low, the process of apoptosis is instructed to occur and autophagy is also induced to prosurvive at the same time. From this point, there may be a balance between them, and we can regulate their interactions to achieve the desired results someday.

In conclusion, we discovered that curcumin effectively inhibited the proliferation of PCa cells by acting on different molecular mechanisms, including arresting PCa cells at G2/M phase, and inducing apoptosis and autophagy. Although curcumin is a monomer, it has a very wide range of effects, which may be one of the reasons for its high anticancer efficacy. Curcumin, therefore, is a potent multitargeted suppressor of PCa cell viability and may become a novel therapeutic candidate for PCa. Based on the finding of the present study that curcumin could induce apoptosis and autophagy, our next work will focus on the underlying mechanisms.

## Figures and Tables

**Table 1:** Relative ratio of Bax and LC3II.

	C10/control	C20/control	C40/control	C80/control
Relative ratio of protein Bax	1.04480417	1.26475652	1.455598125	1.582404383
Relative ratio of protein LC3II	0.79957309	1.60000026	5.737708117	9.365680576
LC3II/Bax	0.76528512	1.26506584	3.941821591	5.918639178

C10, C20, C40, and C80: dosing group with different concentration of curcumin. LC3II/Bax: relative ratio of two above.
